# How AI literacy predicts L2 Chinese writing engagement: the mediating roles of anxiety and enjoyment

**DOI:** 10.3389/fpsyg.2026.1811031

**Published:** 2026-05-08

**Authors:** Chenrui Miao, Jingyao Shi, Jili Jiang

**Affiliations:** 1 The School of Education, Curtin University, Perth, WA, Australia; 2 University International College, Macau University of Science and Technology, Macao, Macao SAR, China; 3 Mental Health Education and Counseling Center of the Student Affairs Office, Hezhou University, Hezhou, China

**Keywords:** AI literacy, L2 anxiety, L2 Chinese learners, L2 engagement, L2 enjoyment

## Abstract

**Introduction:**

In the era of Generative AI, the focus of second language (L2) writing research has shifted toward how learners’ AI literacy shapes their affective experiences and engagement. Drawing upon the Control-Value Theory (CVT), this study investigated the mechanisms underlying the relationship between L2 Chinese Writing AI Literacy and L2 Engagement, with L2 Writing Anxiety and L2 Writing Enjoyment as mediating variables.

**Methods:**

The study employed an explanatory sequential mixed-methods design. In the quantitative phase, structural equation modeling (SEM) was conducted with 408 L2 Chinese learners. In the subsequent qualitative phase, Stimulated Recall Diaries (SRD) and Stimulated Recall Interviews (SRI) were conducted with eight participants to gain an in-depth understanding of their emotional trajectories.

**Results:**

Quantitative results confirmed that L2 Chinese Writing AI Literacy significantly predicted L2 Engagement both directly and indirectly. Specifically, AI literacy served as a powerful affective regulator, significantly reducing L2 anxiety by enhancing learners’ perceived control and thereby boosting writing engagement. Qualitative findings revealed that AI literacy empowered learners to transition from anxious novices to confident editors, where the exercise of selective agency became a primary source of control.

**Discussion:**

Overall, the study highlights the role of L2 Chinese Writing AI Literacy as an emotional buffer for creative agency in technologically enhanced writing environments. The findings offer critical pedagogical implications for fostering AI literacy and developing differentiated scaffolding strategies to optimize L2 Chinese writing development.

## 1 Introduction

Technological development has arguably been the most influential external driver of change in Second Language (L2) pedagogy over the past three decades (An et al., 2023). More recently, the emergence of large language models (LLMs) has begun to fundamentally reshape the contexts, materials, and instructional approaches of Foreign Language Education (FLE) (Annamalai et al., 2023). According to the reports released by the Higher Education Policy Institute (HEPI) (2025) and the Digital Education Council (2024), approximately 92% of students enrolled in higher education worldwide have used generative AI (GenAI) technologies for academic purposes, representing an increase of around 26% compared with the previous year. Notably, about 86% of learners reported that they are currently using, or have previously used, GenAI in creative academic activities such as writing.

Unsurprisingly, this trend has attracted substantial attention from L2 educators and researchers, as it may generate new insights, pedagogical approaches, and learning resources that facilitate higher-quality and more effective L2 writing instruction. L2 writing has long been recognised as one of the most challenging areas for both learners and teachers, partly because it requires learners to deploy multidimensional, higher-order metacognitive writing strategies (Abdel Latif, 2021). In turn, L2 writing students may experience self-doubt, frustration, and even withdrawal, which can lead to a decline in L2 engagement. However, engagement is widely regarded as a key contributor to meaningful learning (Tsao, 2024). As a result, GenAI has the potential to foster more effective learner engagement and to enhance learner autonomy, positive affective experiences, and learning attitudes both inside and outside the classroom (Hyland, 2010).

Over the past decade, the number of learners acquiring Chinese as a second language (L2 Chinese) has grown rapidly, attracting increasing scholarly interest in L2 Chinese pedagogy (Gong and Lai, 2024). L2 Chinese writing is likewise perceived by many learners as one of the most difficult skills to develop, largely because Chinese character writing, lexical density, and syntactic structures differ substantially from those of alphabetic languages (Allen, 2008). In this context, investigating how GenAI can be integrated into dialogic interaction and feedback-based assessment in L2 Chinese writing has become both timely and necessary.

However, recent work on GenAI in L2 Chinese writing pedagogy has largely focused on the development of GenAI-assisted instructional materials and learning platforms, as well as a limited number of studies examining learners’ engagement with GenAI-generated feedback (Li et al., 2025), which has given rise to several potential research gaps. First, there remains a lack of foundational investigation into AI literacy among L2 Chinese learners in GenAI-supported writing contexts. Specifically, existing studies have not sufficiently demonstrated which aspects of AI literacy L2 learners develop most prominently in relation to writing, whether the development of these aspects is balanced, and how widely discussed issues such as AI ethics manifest in the practices and perceptions of L2 Chinese writers. Without such baseline evidence, it becomes difficult to formulate pedagogically actionable and context-sensitive recommendations for the sustainable integration of GenAI in L2 Chinese writing instruction.

Second, the relationship between AI literacy and L2 writing engagement has received limited attention in L2 Chinese writing research. In L2 English education, a growing body of research in recent years has begun to examine how L2 AI literacy relates to positive affective outcomes such as enjoyment in writing (Belda-Medina and Calvo-Ferrer, 2022; Zou et al., 2025). Within this line of inquiry, Control–Value Theory (CVT) has served as a key theoretical framework, as it posits that learners’ emotions during learning activities are shaped by their appraisals of perceived control and task value (Pekrun, 2006). Nevertheless, empirical research adopting this perspective remains scarce in the context of L2 Chinese writing pedagogy.

Third, current research provides insufficient evidence regarding the mediating mechanisms through which AI literacy, as an antecedent variable, may jointly influence learner engagement in L2 writing. CVT further suggests that emotions experienced during learning can significantly shape learners’ subsequent investment and engagement, and that enhancing positive emotions is critical for promoting sustained engagement (Pekrun et al., 2009), which also resonates with what the Self-determination Theory (SDT) proposed concepts of connection, competence and autonomy (Deci and Ryan, 1985).

Accordingly, grounded in CVT, the present study aims to examine the level and developmental profile of AI literacy among L2 Chinese learners in GenAI-supported writing instruction, the association between AI literacy and L2 Chinese writing engagement, and how L2 anxiety and L2 enjoyment jointly mediate this relationship.

## 2 Literature review

### 2.1 AI literacy in L2 Chinese writing

L2 Chinese writing has consistently been regarded in prior research as a learning domain that many L2 Chinese learners tend to resist, which is largely attributable to fundamental differences between the Chinese character production system and the writing systems of most learners’ first languages (Zhao and Wang, 2025). First, Chinese characters cannot be produced through phonetic decoding alone (Bai and Guo, 2021), which means that many L2 Chinese learners encounter substantial obstacles at the initial stages of writing (Du and Chen, 2021). Second, Chinese writing is typically characterised as topic-prominent, whereas languages such as English are subject-prominent (Francis, 2010). As a result, the compositions of many L2 Chinese writers are often described as “English-like Chinese,” characterised by the overuse of explicit connectives or by placing temporal and locative information at the end of sentences (McGinnis, 1999).

However, the emergence of GenAI has offered new possibilities for addressing the challenges outlined above. One particularly salient advantage is its potential to support control in L2 Chinese writing. LLMs like ChatGPT, Gemini, and Bard have completely advanced and renewed the power and capacities of AWE (Ma, 2025). Those chatbots include self-adaptation systems, which tailor all responses and advice to specific users’ prompts and speaking habits (Zou et al., 2025). Furthermore, its circular memory automatically captures users’ usage patterns and past discussions (JunWei et al., 2025), thereby continuing conversations when users use it again (Adeshola and Adepoju, 2023). Its renovations swiftly change attitudes toward automated feedback of the customer base, meeting everybody’s expectations and greatly improving its acceptance (Amer et al., 2025). These capabilities, unique to LLMs, will, in turn, foster confidence and motivation in L2 Chinese writing, as students may believe that with a strong assistant, they can address more issues and challenges in their writing. Even more importantly, all the confusion and fear in writing can be addressed immediately by chatbots, so that students will not doubt that their writing will be doomed to criticism. CVT argues that when an individual believes he can handle more uncertainty in learning activities, he is more likely to experience a sense of achievement (Pekrun, 2006).

Past research in L2 English showed that L2 writers with higher AI literacy are more likely to engage in multi-turn, negotiation-oriented interaction with AI, thereby cultivating an internal drive to develop written language competence (Darmawansah et al., 2024). Bhusal (2025) argued that L2 writers with higher AI literacy can be more proficient at evaluating the quality of AI-generated feedback and incorporating it critically. By contrast, L2 writers with lower AI literacy may become overly dependent on AI feedback, or even rely on AI to generate substantial portions of their texts, increasing the risk of inappropriate use and academic misconduct (Cai et al., 2023).

However, although theoretical discussions in the L2 Chinese field and practice in L2 English have offered substantial insights into the pedagogical potential of GenAI, relatively few empirical studies have investigated L2 AI writing literacy among L2 Chinese learners (Baogui, 2023; Huanyu and Wenjuan, 2024). Huang et al. (2025a) pointed out that AI literacy consists of four dimensions, including understanding, application, ethical cognition and self-efficacy, which to some extent can determine students’ control appraisal and task value appraisal. This raises a critical question: even if LLMs are proven powerful, do L2 Chinese learners actually possess the literacy and skills required to effectively leverage these tools to support their writing development? The next aspect we need to explore further is whether LLMs can foster a sense of achievement when engaging in meaningful negotiation during the writing process.

### 2.2 The synergistic transformation of AI literacy, L2 enjoyment, and L2 anxiety in L2 Chinese writing

In recent years, drawing on educational psychology (Sternberg and Preiss, 2010) and affective theories (Dewaele and MacIntyre, 2019) in L2 research, an increasing number of scholars have shifted attention toward an affective perspective (Li et al., 2025; Acatrinei, 2024). This shift is partly motivated by accumulating evidence that learning environments—particularly pedagogical interaction in classroom contexts—can foster L2 learners’ affective connection with the target language, thereby shaping their learning motivation and language learning performance (Du and Yang, 2025). Among a wide range of emotions, L2 anxiety has often been recognised as a salient affective factor that directly disrupts learners’ attentional focus, reduces classroom participation, and constrains their effective use of the cognitive resources provided by teachers, ultimately undermining the value and effectiveness of L2 instruction (Adeshola and Adepoju, 2023; Huang et al., 2025b).

To alleviate L2 anxiety, earlier empirical studies have examined why L2 learning elicits anxiety and have identified several prominent antecedents, including unfamiliar learning environments (Botes et al., 2020), peer pressure (Acedo et al., 2017), and limited access to effective or timely teacher feedback (Abolhasani et al., 2022). These factors are particularly obvious in L2 writing contexts, where learners’ performance is highly visible and heavily dependent on feedback and revision (Safari and Ahmadi, 2025). More recently, however, research has suggested that although L2 anxiety is typically conceptualised as a negative emotion, it does not necessarily lead to negative outcomes (Zhang, 2019). Rather, its effects may depend on appropriate instructional guidance and pedagogical adaptation (Hiver et al., 2020).

From the perspective of CVT, learners’ achievement emotions are contingent upon their cognitive appraisals of the writing task (Pekrun, 2006). L2 learners may experience heightened anxiety when they perceive a lack of control over the complex linguistic demands of Chinese writing, especially when the task value is high. However, moderate anxiety does not necessarily lead to negative outcomes (Zhang, 2019); rather, it may serve as a driving force that pushes learners to adopt proactive coping strategies and strive toward their learning goals (Yin and Fathi, 2025).

Accordingly, the emergence of GenAI offers a potentially efficient pathway for alleviating and positively regulating L2 writing anxiety (Yang, 2025). Due to its time- and location-independent affordances, GenAI enables L2 learners to engage in dialogue with AI-powered chatbots in familiar environments and at self-selected times, thereby reducing the discomfort associated with unfamiliar learning contexts (Wang et al., 2025). Moreover, because such interaction typically takes place in a private one-to-one setting, L2 writers are less likely to experience performance pressure stemming from social comparison, which may otherwise trigger doubts about their own competence (Aljohani, 2024). In addition, by functioning as an individualized interlocutor, GenAI can provide immediate feedback tailored to learners’ writing preferences and academic profiles, potentially mitigating the sense of pressure that learners may experience in classroom settings when teachers are constrained by limited time or adopt more directive instructional styles (Barrot, 2023a).

During this process, L2 writers may experience an immersive writing state driven primarily by intrinsic motivation in one-to-one writing activities (Barrot, 2023b). At this stage, L2 anxiety no longer dominates learners’ affective experience in writing. Instead, as learners gain increasing control over the writing process and observe continuous improvement in their written output, a positive emotion—L2 enjoyment—emerges and gradually becomes salient (Dewaele and MacIntyre, 2014).

L2 enjoyment represents a salient affective variable in L2 Chinese writing instruction and warrants greater scholarly attention. Historically, influenced by Confucian educational traditions, Chinese pedagogical culture has often embraced the belief that learning is inherently arduous, encapsulated in the notion that “learning is a bitter journey.” Within this view, enjoyment has traditionally been regarded as incompatible with serious learning (Xu et al., 2024). However, Dewaele and Li (2021) demonstrated that L2 enjoyment can broaden learners’ cognitive scope, encourage more flexible thinking, and facilitate creative problem-solving, thereby supporting language learning.

Empirically, Wang et al. (2025) randomly assigned 229 Vietnamese learners of L2 Chinese into three groups and found that higher levels of L2 anxiety were associated with lower levels of task completion in L2 writing. Despite such evidence, research examining the relationships among AI literacy, L2 anxiety, and L2 enjoyment in L2 Chinese writing remains limited, highlighting the need for more systematic investigation.

### 2.3 L2 engagement in L2 Chinese writing

Affective responses are emphasised in L2 writing instruction because affective involvement can, to a considerable extent, influence learners’ willingness to participate in learning activities and sustain task-focused attention, both of which constitute a critical prerequisite for effective learning (Zhou et al., 2022). Philp and Duchesne (2016, p. 15) define engagement as “a state of heightened attention and involvement.” Building on this definition, engagement is widely conceptualised as a multidimensional construct encompassing behavioural, affective, and cognitive dimensions (Berry, 2020). Among these, behavioural engagement has received the greatest attention in L2 pedagogy, while affective and cognitive engagement are generally viewed as complementary dimensions that support observable learning behaviours (Christenson et al., 2022).

A substantial body of prior research has focused on feedback practices in L2 writing instruction. Yao et al. (2024) found that L2 teachers who hold a growth mindset tend to provide more effective written feedback and are better able to tailor their feedback to learners with different needs. Alongside the rapid rise of GenAI, Automated Writing Evaluation (AWE) has also attracted increasing attention from L2 educators. Zhang and Hyland (2018) developed an engagement model by comparing teacher-provided feedback with AWE feedback, demonstrating that learner engagement exhibits dynamic variation across different feedback modes. More recently, Long (2024) reported that although L2 learners generally value feedback generated by AWE systems, they may struggle to interpret certain feedback in the absence of adequate teacher support.

However, engagement in L2 writing extends beyond feedback processing to encompass learners’ sustained attention, effort allocation, strategic thinking, and affective responses throughout the entire writing process—from planning and drafting to self-initiated revision (Dewaele and Li, 2021). In AI-assisted writing contexts, this broader conceptualisation becomes particularly relevant, as learners must not only engage with AI-generated suggestions but also maintain task focus, regulate their own writing behaviours, and navigate affective fluctuations during human–AI interaction (Huang et al., 2025b; Darmawansah et al., 2024).

Within the field of L2 Chinese writing, several preliminary studies have reported findings that are broadly consistent with those observed in L2 English writing research. For example, Wang et al. (2025) found a negative relationship between L2 writing anxiety and L2 engagement in L2 Chinese writing contexts. Nevertheless, the existing literature still leaves several critical issues insufficiently addressed.

First, further research is needed to clarify how writing literacy among Chinese learners influences engagement in L2 Chinese writing under GenAI-supported conditions. Previous studies have suggested that higher levels of AI literacy may enhance L2 enjoyment, promote negotiation-oriented interaction between learners and computational tools, and ultimately improve writing quality (Lyu and Salam, 2025), where AI literacy dimensions, such as applying and understanding, function as critical resources for enhancing learners’ perceived control within the CVT framework (Pekrun, 2006). However, questions remain as to whether learners with lower levels of AI literacy can also experience enjoyment and thereby partially enhance their writing performance. These possibilities warrant closer empirical examination.

Second, it is necessary to test the mediating mechanisms linking L2 Chinese Writing AI Literacy and L2 Engagement Interaction with AI tools often requires learners to mobilise volitional effort, a sense of agency, and perceived choice in order to translate potential competence into observable action, processes that are closely intertwined with affective filtering (Chen, 2024). AI-assisted writing involves intensive interaction while simultaneously offering a high degree of privacy, conditions that are likely to elicit psychological fluctuations during human–AI dialogue (Darmawansah et al., 2024). Consequently, examining affective mediation is essential for explaining why learners with comparable levels of AI literacy may nevertheless display markedly different degrees of engagement in L2 Chinese writing. In addressing these issues, CVT provides a theoretically grounded framework to guide analysis and interpretation.

Third, while previous studies have primarily relied on cross-sectional quantitative designs to examine the correlates of L2 engagement (e.g., Wang et al., 2025), there is a notable lack of research adopting a mixed-method triangulation approach. Existing literature has yet to adequately explain the dynamic, process-oriented nature of how AI literacy shapes affective experiences over time. By combining structural equation modeling with longitudinal qualitative diaries and stimulated recall interviews, the present study aims to provide a more nuanced, explanatory account of the statistical relationships, thereby offering a deeper understanding of the mechanisms underlying human–AI collaboration in L2 Chinese writing.

### 2.4 Theoretical foundation: control–value theory

The core premise of CVT is that individuals’ perceived control over a task and their subjective appraisal of the task’s value jointly shape how meaningful the task is experienced (Pekrun, 2006). When a task is perceived as meaningful, learners are more likely to experience achievement-related positive emotions, which in turn facilitate sustained task engagement and goal-directed action, ultimately enabling learners to derive benefits from task completion (Li et al., 2025).

CVT comprises three core components: control appraisal, intrinsic value appraisal, and extrinsic value appraisal (Pekrun, 2006). Control appraisal refers to individuals’self-evaluation of their competence in accomplishing academic tasks, as well as their expectations, monitoring, and regulation of anticipated outcomes (Pekrun et al., 2009). In GenAI-supported L2 Chinese writing, it will be presented as an understanding of AI and proper cognitions of AI ethical lines. Students must realise what AI can do, what AI cannot do and what AI can do but should not do (Liu and Wang, 2024). Otherwise, AI may have the opposite effect on L2 Chinese writing (Zhang and Hyland, 2018). Furthermore, students also need to build up a concept of AI service, which means AI should be a tool and a servant for improving students’ writing performance, and students can initiate different prompts and continuously polish their prompts to require AI to output more valuable content but not wait for a static AI answer and take them all (Huang et al., 2025a). Intrinsic value appraisal primarily concerns learners’ affective and value-based perceptions of the task itself (Pekrun et al., 2002), which signifies the application of literacy in interactions with AI Once a sufficient sense of control has been established, L2 learners may gradually enter a state of flow (Li and Dewaele, 2024), characterised by immersive and optimal psychological engagement in higher-order interaction with AI. In this phase, L2 Chinese writers are likely to perceive and evaluate GenAI from the perspectives of collaborators and evaluators, critically examining how AI supports the construction of writing-related thinking, the refinement of initial drafts, and the provision of more realistic, usable, and valuable writing content, perspectives, and multi-angled evaluations of the same writing topic. As a result, learners often experience achievement-related academic emotions during this process. Extrinsic value appraisal, by contrast, is outcome-oriented and refers to learners’evaluation of whether task engagement yields observable and quantifiable instrumental gains (Pekrun and Linnenbrink-Garcia, 2012). At this stage, after experiencing control tasks and deeper, more informative interactions with AI, students will form attributions about self-efficacy, an important aspect of AI literacy (Dewaele and MacIntyre, 2014).

Within GenAI-supported L2 Chinese writing contexts, CVT conceptualises AI literacy as a personal resource. Through the development of perceived control, AI literacy helps learners mitigate anxiety during the writing process while facilitating the appraisal of intrinsic task value, thereby enhancing L2 enjoyment. As anxiety-related avoidance-oriented disengagement is reduced and enjoyment-related expansive engagement is fostered, L2 Chinese writers are ultimately able to attain greater extrinsic task value, leading to higher overall levels of engagement in L2 writing tasks (Pekrun and Linnenbrink-Garcia, 2012).

### 2.5 Current study

The present study adopts an explanatory sequential mixed-methods design to investigate the complex and dynamic relationship between AI literacy and L2 Chinese writing engagement. In the first phase of the study, covariance-based structural equation modeling (CB-SEM) approach is employed to examine the hypothesised relationships among the focal constructs. Based on the theoretical framework outlined above, the following hypotheses are proposed:

*H1*: L2 Chinese Writing AI Literacy has a significant positive effect on L2 Chinese Writing Engagement.

*H2*: L2 Writing Anxiety mediates the relationship between L2 Chinese Writing AI Literacy and L2 Chinese Writing Engagement.

*H2a*: L2 Chinese Writing AI Literacy is negatively associated with L2 Writing Anxiety.

*H2b*: L2 Writing Anxiety is negatively associated with L2 Chinese Writing Engagement.

*H3*: L2 Writing Enjoyment mediates the relationship between L2 Chinese Writing AI Literacy and L2 Chinese Writing Engagement.

*H3a*: L2 Chinese Writing AI Literacy is positively associated with L2 Writing Enjoyment.

*H3b*: L2 Writing Enjoyment is positively associated with L2 Chinese Writing Engagement.

*H4*: L2 Writing Anxiety is negatively associated with L2 Writing Enjoyment.

Structural equation modelling (SEM), grounded in rigorous statistical analysis and large-scale datasets, is well suited to uncovering complex associations among variables and estimating path coefficients and predictive power (Hair, 2009). However, SEM is inherently limited to capturing relationships at a single cross-sectional snapshot of a dynamic system (Bieleke et al., 2021). According to CVT, learners’ emotions in L2 learning are inherently dynamic rather than static; moreover, anxiety and enjoyment tend to fluctuate over time and alternately dominate learners’ cognitive processing and adaptive functioning in L2 contexts (Pekrun and Perry, 2014).

To address these limitations and to complement the quantitative model, the present study incorporates a small-sample exploratory–explanatory sequential qualitative longitudinal design. In the second phase, data are collected over a four-week period through stimulated-recall L2 Chinese writing journals and semi-structured interviews, allowing for in-depth engagement with learners’ everyday writing practices. This qualitative phase aims to examine how L2 Chinese learners subjectively experience and agentively employ AI literacy in their writing processes, and how they perceive its influence on L2 writing engagement. In addition, particular attention is given to how L2 writing anxiety and L2 writing enjoyment operate in distinct ways as mediating emotional mechanisms between L2 Writing AI Literacy and L2 Writing Engagement.

Accordingly, the following research questions are proposed for the second phase of the study:

RQ1: How do L2 Chinese learners’ subjective experiences of L2 Writing AI Literacy and their agentive use of AI tools explain their levels of engagement in L2 writing?
RQ2: How do L2 Writing Anxiety and L2 Writing Enjoyment account for the emotion-driven mediating processes between L2 Writing AI Literacy and L2 Writing Engagement?

## 3 Method

The study adopts a mixed-methods case study approach, as Mixed-Methods Research (MMR) ‘enables a more comprehensive understanding of phenomena than single-method approaches by combining particularity with generality, patterned regularity with contextual complexity, while integrating both emic and etic perspectives’ (Cohen et al., 2002, p.33). The research consists of two distinct phases: Study 1 (Quantitative) employs Structural Equation Modeling (SEM) to test the proposed hypothesizes (*N* = 408). Building on the statistical results, Study 2 (Qualitative) utilizes a longitudinal diary approach and stimulated recall interviews with a purposive sub-sample (*N* = 8) to provide a nuanced, process-oriented explanation of the quantitative findings.

### 3.1 Participants

Data were collected via an online survey distributed to L2 Chinese learners from five comprehensive universities in Chinese universities through a convenience sampling strategy. A total of 450 questionnaires were initially returned. Following rigorous data screening, 42 responses were excluded due to the failure to pass the attention-check items, resulting in a valid sample of 408 participants.

**Figure 1:**
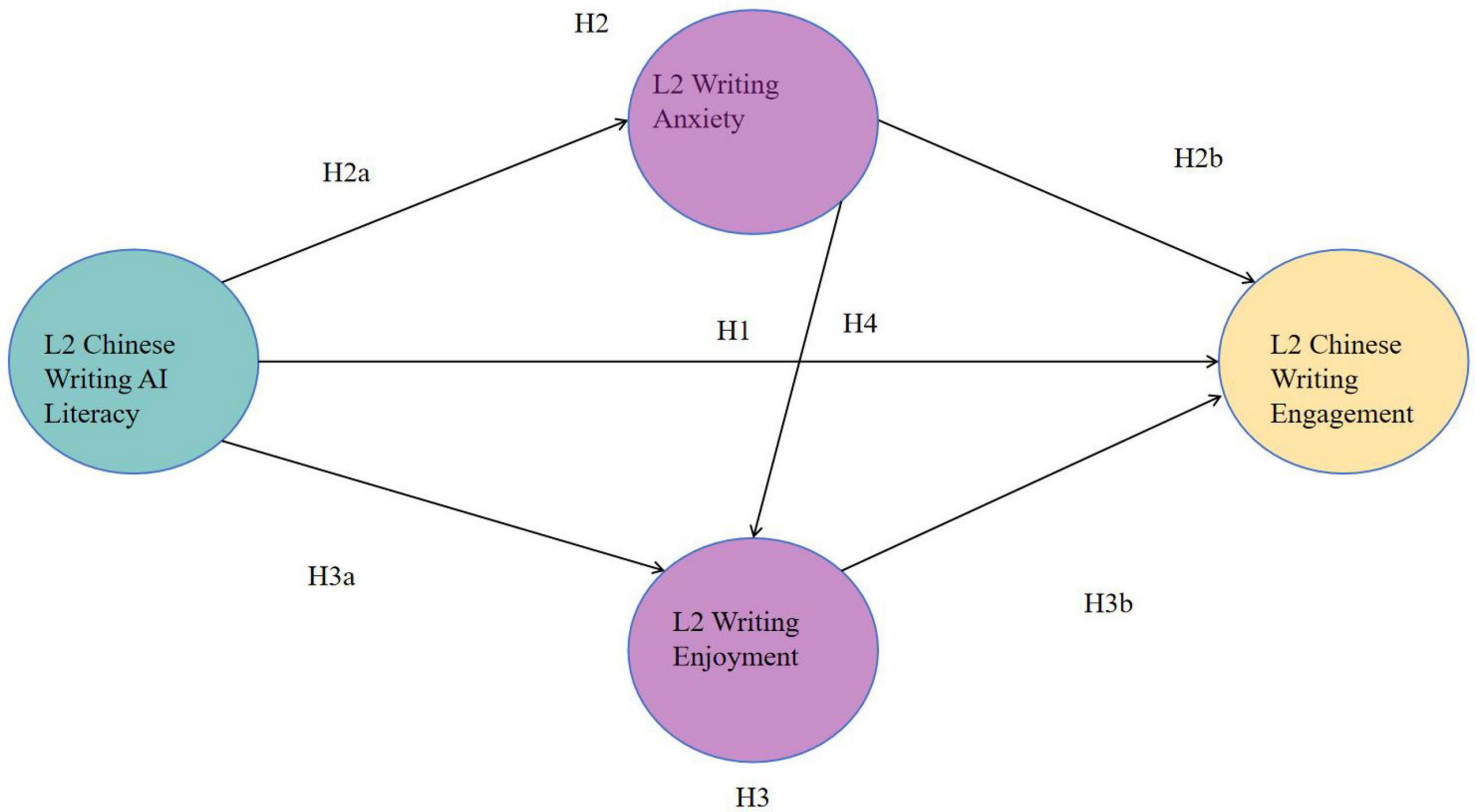
Proposed SEM model about L2 Chinese writing AI literacy and L2 Chinese writing engagement.

All participants provided digital informed consent, and anonymity was strictly maintained throughout the data collection process. Figure 1 presents the demographic profile of the participants (Table 1).

**Table 1:** Basic demographic information of 366 participants.

Demographic information		Amount (*n* = 408)	Percentage
Age	18–19 years	100	24.5%
	19–20 years	127	31.1%
	20–21 years	91	22.3%
	21 + years	90	22.1%
Sex	Female	351	86.0%
	Male	57	14.0%

For the second phase, a purposive sampling strategy—specifically, maximum variation sampling (Patton, 2015)—was employed to select 8 participants from the Study 1. Selection was based on their willingness to participate in the next study.

To control for potential confounding variables, participants were selected from a pool of 28 volunteers who had attained HSK Level 3 or Level 4 proficiency. At this proficiency stage, most Chinese universities formally introduce systematic writing instruction for international students. Selecting learners at these two adjacent proficiency levels therefore allows for the minimisation of variability attributable to proficiency-related factors, thereby reducing the influence of extraneous variables on the focal constructs examined in the present study.

In addition, the study sought to include learners with diverse profiles, such as those exhibiting high versus low levels of anxiety and high versus low levels of AI literacy, and to incorporate participants from different national backgrounds in order to obtain a more comprehensive perspective. Ultimately, eight learners were purposively selected to participate in the second phase of the study.

Detailed demographic profiles of the eight participants (coded as P1 through P8) are presented in Table 2. All participants agreed to engage in a four-week longitudinal observation involving weekly diaries and follow-up interviews.

**Table 2:** Demographic information of 8 students taking part in the stimulated recall dairies and interview.

Number coding	Education	Gender	Chinese proficiency	Nationality
Student 1 (S1)	Undergraduate	Female	HSK-4	South Korea
Student 2 (S2)	Undergraduate	Female	HSK-4	Russia
Student 3 (S3)	Undergraduate	Male	HSK-3	France
Student 4 (S4)	Undergraduate	Female	HSK-3	Brazil
Student 5 (S5)	Undergraduate	Male	HSK-4	The Arab Republic of Egypt
Student 6 (S6)	Undergraduate	Female	HSK-4	Australia
Student 7 (S7)	Undergraduate	Female	HSK-4	Malaysia
Student 8 (S8)	Undergraduate	Male	HSK-3	United States

### 3.2 Instruments

#### 3.2.1 Quantitative instruments (study 1)

To measure AI literacy in L2 writing, the present study adapted the AI Literacy in L2 Writing Scale (AIL-L2WS) developed by Huang et al. (2025a). The scale comprises four dimensions: Understanding AI in L2 Writing (UAI), Applying AI in L2 Writing (AAI), Ethics and Risk Awareness (ERA), and Self-Efficacy and Attitudes (SEA). The original scale demonstrated excellent internal consistency, with a Cronbach’s alpha of 0.960.

To ensure contextual alignment with the current study, all references to English writing were revised to Chinese writing. The full set of 26 items identified in the original exploratory phase was retained. At the same time, particular attention was paid to the four items flagged in the original study’s confirmatory factor analysis (CFA), which were carefully evaluated for potential exclusion based on model fit indices in the present analysis.

To measure L2 writing enjoyment, the present study adapted the short form of the Achievement Emotions Questionnaire (AEQ-S) developed by Bieleke et al. (2021). Specifically, four items from the learning-related enjoyment subscale were adapted to fit the context of L2 Chinese writing. For example, the original item “I look forward to studying” was modified to “I look forward to working on my Chinese writing tasks.” This adaptation ensured that the scale captured activity-specific enjoyment associated with the writing process, as postulated by CVT. The original scale demonstrated satisfactory internal consistency, with a Cronbach’s alpha of 0.82 in the learning context.

To measure L2 writing anxiety, the present study likewise adapted relevant items from the AEQ-S. The original four items were contextualised to reflect anxiety specifically associated with the productive nature of L2 writing. For example, the item “I worry that I will not be able to master all the material” was adapted to “I worry that I will not be able to express my ideas clearly in Chinese.” This modification aligns with prospective outcome anxiety as defined within CVT, focusing on learners’ fear of failure in the specific task of language production.

To measure L2 writing engagement, the present study adapted the scale developed by Hiver et al. (2020). The original instrument was designed to assess engagement in general language classroom contexts. Consistent with the context-specificity principle in engagement research, all items were adapted to target engagement specifically within L2 Chinese writing contexts.

The adapted scale comprises 15 items across three sub-dimensions: Behavioural Engagement (e.g., effort and persistence in writing tasks), Emotional Engagement (e.g., enthusiasm for writing activities), and Cognitive Engagement (e.g., the use of deep processing strategies such as connecting AI-generated feedback with prior knowledge). Negatively worded items (e.g., “I do just enough to finish the writing task”) were reverse-coded prior to analysis. The original scale demonstrated high reliability, with McDonald’s *ω* exceeding 0.86 across all dimensions.

The full questionnaire is provided in Supplementary materials.

#### 3.2.2 Qualitative instruments (study 2)

In the qualitative phase, data collection was implemented in two stages. This design enabled within-phase triangulation through multiple qualitative sources and also facilitated cross-phase triangulation with the quantitative findings from Phase 1.

In the first qualitative stage, the study employed a four-week structured diary to document how the eight participants used AI to guide and support their L2 Chinese writing both inside and outside the classroom. The diary entries were completed on an ongoing basis, allowing participants to record their learning experiences and capture their psychological and affective fluctuations across the four-week period in a timely manner.

To capture the micro-genetic development of learners’ affective and cognitive states, a Structured Digital Diary protocol was employed over the four-week intervention period. Unlike unstructured journals, these prompts were theoretically anchored in the CVT (Pekrun, 2006). The prompts were designed to elicit daily reflections on specific interactional episodes with AI tools, focusing on dynamic fluctuations in Control Appraisals, Value Appraisals, and the resulting Emotional and Behavioral Engagement.

Prior to implementing the diary protocol, the researcher held a brief introductory conversation with each of the eight invited participants to gain an initial understanding of their learning backgrounds, learning dispositions, and perceptions of Chinese writing. On this basis, the weekly diary prompts were refined and organised into four sequential stages. Each week centred on one overarching theme and included three guiding questions for participants to address.

The Week 1 theme was designed to capture participants’ initial manifestations of AI literacy and establish a baseline of their affective fluctuations while using AI during the writing process. Week 2 focused on how AI literacy shaped learners’ engagement in L2 Chinese writing. Week 3 examined how L2 enjoyment and L2 anxiety were perceived to influence L2 engagement. Week 4 explored how learners perceived AI literacy as reshaping their learning attitudes and self-concept. In total, the four-week diary protocol comprised 12 questions, which are provided in Supplementary materials.

Participants were asked to complete one diary entry each Friday. They were instructed not merely to report what they had done, but to foreground their in-the-moment emotions (e.g., anxiety, enjoyment, frustration, surprise) and the thoughts accompanying those feelings. Diary entries were submitted via WeChat or email to ensure ease of access for these “digital natives.” Participants were encouraged to write in their preferred language to maximise the richness and accuracy of their emotional expression.

In the second qualitative stage, stimulated recall interviews (SRIs) were conducted using the textual data collected in Stage 1 as the basis for eliciting participants’ retrospective accounts. Through guided dialogue, the SRIs aimed to reconstruct writers’ moment-by-moment experiences and, through triangulation between diary records and interview accounts, provide explanatory insights into the theorised mechanisms underlying the quantitative model. Following Dempsey (2010), the SRIs used textual artefacts—including original drafts, AI interaction logs, and final revisions—as visual stimuli to reactivate participants’ cognitive processes.

To ensure comparability with the Phase 1 SEM model, six semi-structured interview questions were developed. These questions began with participants’ overall experiences and then progressively probed emotional mechanisms and engagement processes, ultimately addressing issues of identity/agency and anticipated future behaviour. The full interview protocol is provided in Supplementary materials.

### 3.3 Data analysis

#### 3.3.1 Quantitative analysis

Quantitative data analysis was conducted in two phases using SPSS 31.0 and AMOS 31.0. First, data screening was performed to check for missing values, outliers, and normality. Descriptive statistics were calculated to summarize participants’ demographic profiles and item responses. To assess the reliability and validity of the measurement model, CFA was conducted. Construct reliability was evaluated using Cronbach’s *α* and Composite Reliability (CR), while convergent validity was assessed via Average Variance Extracted (AVE).

Second, CB-SEM was employed to test the hypothesized relationships among AI Literacy in L2 Writing, L2 anxiety, L2 enjoyment, and L2 engagement. Model fit was evaluated based on established indices: χ^2^/df < 3.0, CFI > 0.90, TLI > 0.90, RMSEA < 0.08, and SRMR < 0.08 (Hu and Bentler, 1999). Finally, to test the mediating roles of L2 anxiety and enjoyment, a bias-corrected bootstrapping method (with 5,000 resamples) was utilized to determine the significance of indirect effects at the 95% confidence interval.

#### 3.3.2 Qualitative analysis

Qualitative data, comprising structured diaries collected over four weeks and eight stimulated recall interview transcripts, were analysed using thematic analysis following the six-phase framework proposed by Braun and Clarke (2006). Following Braun and Clarke (2006), the analysis proceeded in six phases. All analyses were conducted in NVivo 15.

Firstly, the researchers immersed themselves in the data by reading and re-reading the transcripts to note initial ideas. Secondly, NVivo 15 was used for coding. Initial codes were systematically generated across the entire dataset (e.g., “fear of errors,” “AI as a partner”). Thirdly, the codes were collated into potential themes, specifically looking for patterns related to control–value appraisals and emotional regulation. Fourthly, the themes were reviewed against the coded extracts and the entire dataset to ensure internal homogeneity and external heterogeneity. Fifthly, themes were refined to generate clear definitions and names (e.g., “From Threat to Tool: Re-establishing Control”). Sixthly, compelling extract examples were selected to relate back to the research questions and literature.

To ensure trustworthiness, two researchers independently coded a subset of the data (20%). Intercoder agreement was calculated (Cohen’s kappa > 0.80), and discrepancies were resolved through discussion.

## 4 Results

### 4.1 Descriptive statistics

Table 3 presents the descriptive statistics for the 408 participants across the key constructs of the study, including AI Literacy, L2 Writing Anxiety, L2 Writing Enjoyment, and L2 Engagement.

**Table 3:** Descriptive statistics for key constructs.

Construct	*N*	Mean	Std. deviation	Skewness		Kurtosis	
	Statistics	Statistics	Statistics	Statistics	Std. Error	Statistics	Std. error
Understanding AI in L2 writing	408	3.31	1.42	−0.273	0.121	−1.274	0.241
Applying AI in L2 writing	408	3.29	1.39	−0.262	0.121	−1.226	0.241
Ethics and risk awareness	408	3.27	1.41	−0.252	0.121	−1.287	0.241
Self-efficacy and attitudes	408	3.32	1.4	−0.266	0.121	−1.276	0.241
L2 Chinese writing AI literacy	408	3.3	1.41	−0.263	0.121	−1.266	0.241
L2 Enjoyment	408	3.2	1.34	−0.17	0.121	−1.129	0.241
L2 Anxiety	408	2.89	1.33	0.121	0.121	−1.192	0.241
Behavioral engagement	408	3.3	1.38	−0.283	0.121	−1.191	0.241
Emotional engagement	408	3.28	1.36	−0.242	0.121	−1.181	0.241
Cognitive engagement	408	3.29	1.37	−0.251	0.121	−1.193	0.241
L2 Engagement	408	3.29	1.37	−0.259	0.121	−1.188	0.241
Valid N (listwise)	408						

The Mean values across most variables range from 2.83 to 3.43 (on a 5-point Likert scale), indicating that, on average, participants hold a generally moderate-to-positive attitude toward AI-assisted learning. Specifically, constructs related to AI Literacy and L2 Engagement received relatively higher ratings (mostly above 3.30), suggesting that students generally perceive themselves as capable of using AI and are engaged in the writing process. In contrast, L2 Writing Anxiety (Items Q31–Q34) showed slightly lower mean scores (ranging from 2.83 to 2.96), implying that while anxiety exists, it is not overwhelmingly high for the average student.

The Standard Deviations (SD), ranging primarily between 1.30 and 1.48, highlight a notable variability in responses. This indicates that the participants’ experiences and proficiency levels are quite heterogeneous; while the average student is positive, there is a wide dispersion of attitudes ranging from highly anxious/disengaged to highly confident/engaged.

Regarding the distribution of the data, the Skewness values (mostly between −0.35 and 0.20) and Kurtosis values (mostly between −1.0 and −1.3) fall well within the acceptable thresholds for Structural Equation Modeling (Skewness < |3.0| and Kurtosis < |7.0|) (Kline, 2011). Although the negative kurtosis values suggest a slightly platykurtic distribution (flatter than a normal curve), the data does not violate the assumption of normality required for subsequent maximum likelihood estimation in AMOS.

### 4.2 Measurement model assessment

CFA was conducted using AMOS 31 to evaluate the reliability and validity of the measurement model. The model fit indices indicated an excellent fit to the data: χ^2^/df = 1.597 (< 3.0), CFI = 0.966, TLI = 0.964, and RMSEA = 0.038.

Convergent validity was assessed by examining the standardized factor loadings, Composite Reliability (CR), and Average Variance Extracted (AVE). As shown in Table 4, all standardized factor loadings ranged from 0.761 to 0.872, exceeding the recommended threshold of 0.50 (Hair et al., 2006). Furthermore, the CR values for all constructs ranged from 0.897 to 0.975, well above the 0.70 cutoff, and all AVE values exceeded the 0.50 threshold, demonstrating strong convergent validity.

**Table 4:** Reliability and validity of the proposed model.

Construct	Item	Factor loading	Cronbach’s α	CR	AVE
Understanding AI in L2 Writing	Q1	0.838	0.898	0.9	0.691
	Q2	0.820			
	Q3	0.798			
	Q4	0.868			
Applying AI in L2 writing	Q5	0.834	0.947	0.95	0.732
	Q6	0.860			
	Q7	0.821			
	Q8	0.802			
	Q9	0.866			
	Q10	0.818			
	Q11	0.798			
Ethics and risk awareness	Q12	0.837	0.932	0.938	0.751
	Q13	0.831			
	Q14	0.810			
	Q15	0.855			
	Q16	0.865			
Self-efficacy and attitudes	Q17	0.865	0.973	0.975	0.796
	Q18	0.862			
	Q19	0.839			
	Q20	0.863			
	Q21	0.835			
	Q22	0.858			
	Q23	0.842			
	Q24	0.845			
	Q25	0.842			
	Q26	0.863			
L2 Enjoyment	Q27	0.872	0.896	0.897	0.686
	Q28	0.833			
	Q29	0.761			
	Q30	0.843			
L2 Anxiety	Q31	0.842	0.901	0.901	0.694
	Q32	0.835			
	Q33	0.806			
	Q34	0.848			
L2 behavioral engagement	Q35	0.847	0.923	0.925	0.712
	Q36	0.846			
	Q37	0.798			
	Q38	0.814			
	Q39	0.809			
L2 emotional engagement	Q40	0.809	0.890	0.893	0.626
	Q41	0.793			
	Q42	0.764			
	Q43	0.791			
	Q44	0.784			
L2 cognitive engagement	Q45	0.794	0.902	0.903	0.651
	Q46	0.814			
	Q47	0.814			
	Q48	0.778			
	Q49	0.756			

Discriminant validity was evaluated using the Fornell-Larcker criterion. As presented in Table 5, the square root of the AVE for each construct was greater than the inter-construct correlations (off-diagonal elements), confirming that the constructs are empirically distinct from one another (Fornell and Larcker, 1981).

**Table 5:** AVE’s square root and inter-construct correlations.

Construct	1	2	3	4
1. L2 Chinese writing AI literacy	0.831			
2. L2 Writing engagement	0.704	0.844		
3. L2 Writing anxiety	−0.643	−0.575	0.833	
4. L2 Writing enjoyment	0.698	0.636	−0.647	0.831

### 4.3 Structural model testing

**Figure 2:**
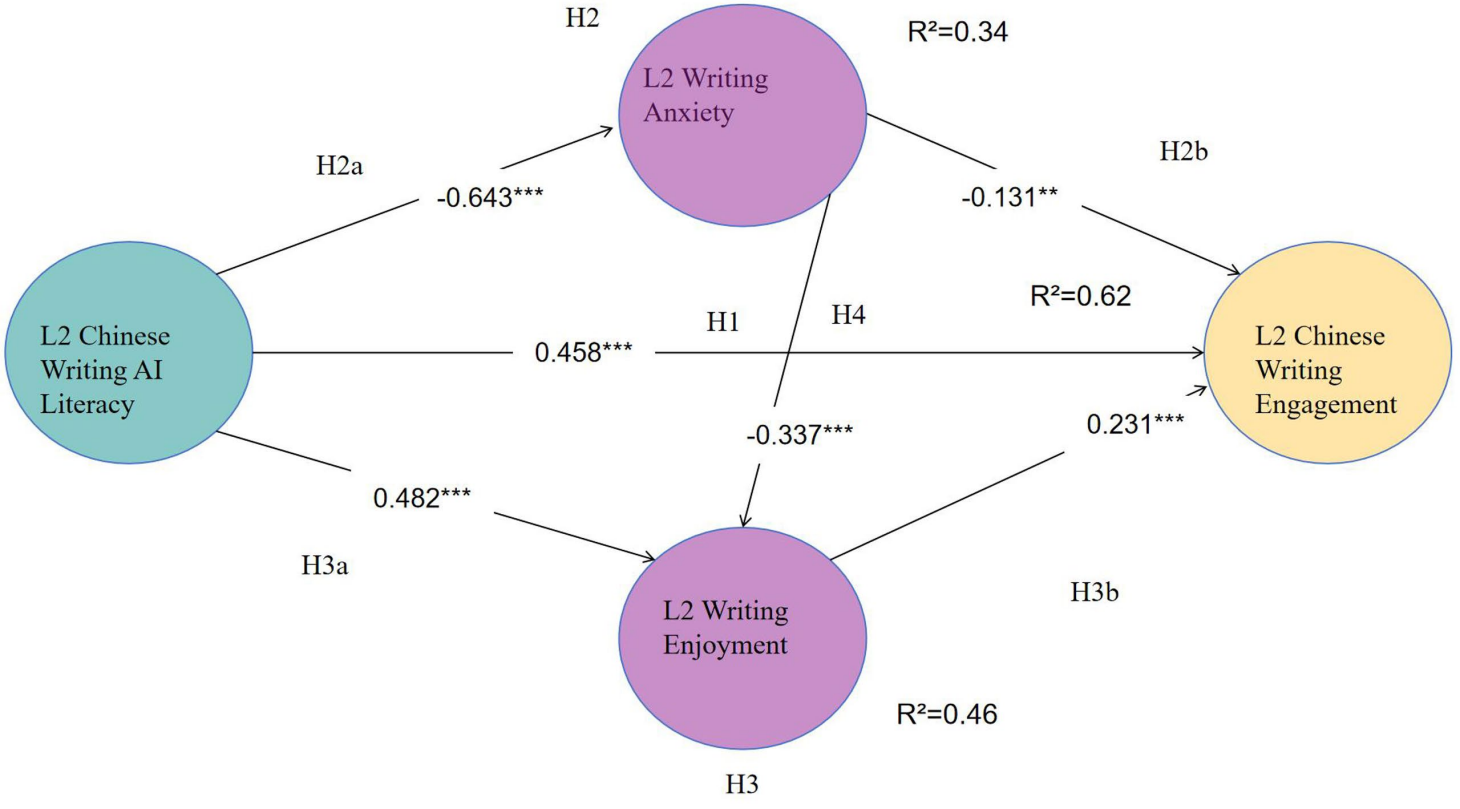
Structural equation model results with standardized path coefficients. *** *p* < 0.001, ** *p* < 0.01, * *p* < 0.05.

Figure 2 illustrates the structural model with standardized path coefficients and the variance explained (R^2^) for each endogenous variable.

AI Literacy was found to be a significant predictor of all outcome variables. Specifically, AI Literacy had a strong negative effect on L2 Writing Anxiety (*β* = −0.643, *p* < 0.001), supporting H2a. This suggests that students with higher AI literacy experience significantly less anxiety. Conversely, AI Literacy positively influenced L2 Writing Enjoyment (*β* = 0.482, *p* < 0.001), supporting H3a. Most importantly, AI Literacy demonstrated a direct positive impact on L2 Engagement (*β* = 0.458, *p* < 0.001), supporting H1.

Furthermore, the results highlighted L2 Writing Anxiety significantly negatively predicted L2 Writing Enjoyment (*β* = −0.337, *p* < 0.001), supporting H4. Furthermore, L2 Writing Anxiety had a negative effect on L2 Writing Engagement (*β* = −0.131, *p* < 0.05), supporting H2b, while Enjoyment positively predicted Engagement (*β* = 0.231, *p* < 0.001), supporting H3b.

The model explained a substantial amount of variance in the endogenous constructs. As shown in Figure 2, the model accounted for 34% of the variance in Anxiety (R^2^ = 0.34), 46% in Enjoyment (R^2^ = 0.46), and 62% in Engagement (R^2^ = 0.62), indicating a high explanatory power of the proposed model.

The hypothesis testing results, derived from the path analysis, are detailed below and summarized in Table 6.

**Table 6:** Hypothesis testing results.

Hypothesis	Structural path	Std. Beta (β)	Unstd. Beta (B)	S. E.	*t*-value (C. R.)	*p*-value	Result
H1	AI Literacy → L2 Engagement	**0.458**	0.447	0.057	7.768	***	Supported
H2a	AI Literacy → L2 Anxiety	**−0.643**	−0.584	0.046	−12.743	***	Supported
H2b	L2 Anxiety → L2 Engagement	**−0.131**	−0.140	0.059	−2.365	0.018	Supported
H3a	AI Literacy → L2 Enjoyment	**0.482**	0.457	0.052	8.756	***	Supported
H3b	L2 Enjoyment → L2 Engagement	**0.231**	0.237	0.062	3.842	***	Supported
H4	L2 Anxiety → L2 Enjoyment	**−0.337**	−0.352	0.058	−6.111	***	Supported

***p < 0.001. Std. Beta = standardized path coefficient; Unstd. Beta = unstandardized path coefficient; S. E. = standard error; C. R. = critical ratio. Bold values indicate path coefficients that are statistically significant (*p* < 0.05 or lower).

To further investigate the mediating mechanisms, a bias-corrected bootstrapping procedure with 5,000 resamples was performed. As summarized in Table 7, the total indirect effect of AI Literacy on L2 Engagement was statistically significant (*β* = 0.245, 95% CI [0.142, 0.335]). Specifically, three significant indirect pathways were identified: (1) via L2 writing anxiety (*β* = 0.084, *p*<0.01), (2) via L2 writing enjoyment (*β* = 0.111, *p*<0.001), and (3) a sequential mediation path from anxiety to enjoyment (*β* = 0.050, *p*<0.01). Notably, since the direct path from AI Literacy to L2 Engagement remained significant (*β* = 0.458, *p*<0.001) after accounting for these mediators, the results confirm a partial mediation model. This suggests that while AI Literacy has a robust direct impact on writing engagement, its influence is also significantly transmitted through the dual affective-regulatory mechanisms of reducing anxiety and boosting enjoyment.

**Table 7:** Standardized indirect effects and 95% confidence intervals.

Mediation path	Effect (*β*)	S. E.	Bootstrapped 95% CI lower	Bootstrapped 95% CI upper	Result
Path 1: AI Literacy → L2 Anxiety → L2 Engagement	0.084	0.035	0.013	0.150	Significant
Path 2: AI Literacy → L2 Enjoyment → L2 Engagement	0.111	0.031	0.048	0.169	Significant
Path 3: AI Literacy → Anxiety → Enjoyment → Engagement	0.050	0.016	0.018	0.079	Significant
Total indirect effect	0.245	0.049	0.142	0.335	Significant

### 4.4 Qualitative findings

#### 4.4.1 The role of AI literacy: from passive reception to active editorial agency

Qualitative data revealed that L2 Chinese Writing AI Literacy manifested primarily as an active editorial and planning capability rather than passive reliance. Participants generally viewed AI as a “scaffolding partner” that reduced the cognitive load. Furthermore, high AI literacy appeared to amplify cognitive engagement.

Participants described a process of “critical comparison” where AI-generated outputs served as evaluative reference for self-correction. For instance, P1 (Korea), a self-identified perfectionist, noted that AI did not induce dependency but rather fostered a habit of deeper reflection. She described her interaction with AI as a recursive cycle of writing, requesting AI revision, and comparing versions:

Excerpt 1: “I spend more time thinking now… I wouldn’t dare use AI’s content directly. I write first, then ask AI to revise. AI gives me many versions, and I keep comparing to see which is more accurate… I hope every revision brings me closer to ‘correct’ and ‘good’.” (P1)

This excerpt illustrates that AI literacy involves the ability to discern and negotiate with AI’s “standardized language.” This iterative cycle of self-correction reflects an enhanced control appraisal over the writing process.

Furthermore, participants maintained a clear boundary regarding authorship agency. P4 (France) articulated a sophisticated understanding of the human-AI division of labor. While acknowledging AI’s linguistic proficiency, she firmly positioned herself as the primary architect of the content:

Excerpt 2: “If I must define it, I would say it is a cautious cooperation. I am the creator of the content; AI is the interpreter of the expression.” (P4)

These findings suggest that L2 Chinese writing AI literacy empowers students to transition from anxious novices to confident “editors,” using AI to bridge the gap between their intended meaning and linguistic execution.

#### 4.4.2 Mitigating L2 writing anxiety: AI as a cognitive and affective buffer

Qualitative findings further elucidated the mechanism by which AI Literacy alleviates L2 writing anxiety, as hypothesized in H2a. Participants characterized AI as a “brainstorming partner” that transformed writing from a solitary, high-stakes task into a collaborative, iterative process.

For participants with higher AI literacy, AI served as a cognitive tool to diversify linguistic expression and overcome the “fear of the blank page.” This interaction was not merely about generating text but about expanding conceptual possibilities. As one participant noted:

Excerpt 3: “For me, this ‘brainstorming’ interaction makes me write more, not lazily. But this ‘more’ isn’t about word count; it’s about having more ideas, more versions, and more attempts.” (P3)

However, the data also highlighted that technical anxiety can coexist with L2WA for learners with lower AI literacy. P4 initially experienced confusion and a sense of “linguistic alienation” when faced with AI’s highly formal output. She expressed a typical affective barrier—the fear of appearing “inauthentic” or losing control over the register:

Excerpt 4: “If I use AI, it might make sentences sound more like formal written Chinese, but that confuses me. The Chinese is sometimes too ‘formal’… I’m not sure if the teacher will think it’s strange.” (P4)

After receiving targeted instruction on prompt engineering and value-reconstruction, her perception of AI shifted from an “external pressure” to an “optional resource.” This psychological shift is crucial for reducing L2 writing anxiety:

Excerpt 5: “I didn’t change from ‘not trusting AI’ to ‘trusting it completely.’ Instead, I moved from ‘AI is definitely not for me’ to ‘certain uses might be helpful.’ This shift… makes learning Chinese feel like having an extra choice rather than extra pressure.” (P4)

These qualitative accounts suggest that AI Literacy mitigates anxiety not by simplifying the task, but by providing a safety net that allows learners to experiment with the language without the immediate fear of “irreversible errors.”

#### 4.4.3 Cultivating L2 writing enjoyment: mindset growth with discovery of task intrinsic value appraisal improved task control

The qualitative data provided rich evidence for H3a, illustrating how AI Literacy fosters L2 writing enjoyment through spurring students’ internal identification of the writing task. Participants reported that interacting with AI was not merely a corrective process but an aesthetic one, allowing them to uncover novel and “authentic” Chinese expressions that were often absent from traditional classroom settings. P6 shared a transformative experience where AI acted as a catalyst for linguistic creativity rather than a source of “copy-paste” content:

Excerpt 6: “I originally wrote: ‘I want people to understand the real China through my videos.’ It felt a bit plain. I asked AI to make it more ‘vivid’… It suggested: ‘through lenses and words (镜头和文字).’ I loved that. It made me realize expression could be so specific. I didn’t copy it all, but I kept ‘lenses.’ I was truly excited. I started trying to change other sentences… I wrote for half an hour longer than usual because I was ‘playing’ with language.” (P6)

This “playfulness” directly supports H3b, demonstrating how the positive affect generated by AI-assisted discovery leads to increased behavioral engagement. The discovery of novel expressions via AI fuels intrinsic value appraisal, which triggers L2 writing enjoyment.

Furthermore, the data revealed that enjoyment is deeply rooted in perceived control and selective uptake.

Participants expressed a sense of achievement not from AI’s proficiency, but from their own ability to negotiate with the machine. P5 articulated this delicate balance of power, describing how the act of “choosing” or even “rejecting” AI suggestions became a source of confidence:

Excerpt 7: “If I accept AI completely, I feel anxious; if I reject it completely, I become rigid. In ‘choice’, I find balance… Rejecting an AI suggestion doesn’t make me feel ‘better than AI,’ but it confirms that I am still the one in charge.” (P5)

The selective appropriation of AI’s feedback reinforces the learner’s responsibility and autonomy, which were identified as the primary drivers of confidence and subsequent enjoyment. By treating AI as a “sounding board” rather than an oracle, learners converted a potentially threatening technology into a source of creative empowerment.

#### 4.4.4 The relationship between task control and engagement: AI literacy as a catalyst for ownership and anxiety reduction

The final theme emerged from the participants’ nuanced understanding of agency and authorship, particularly regarding how AI Literacy mediates the relationship between identity and writing engagement. While participants across all backgrounds viewed AI as a “foundational tool,” their engagement was deeply rooted in the belief that they can take control of AI to facilitate real linguistic performance based on their learning status quo.

For heritage learners (HLs), AI served as a critical bridge between their oral proficiency and formal written requirements. HLs often face a unique form of “identity-related anxiety” stemming from the gap between their cultural heritage and linguistic execution. P7 (Malaysia), a heritage learner, illustrated how AI Literacy allowed her to transition from self-doubt to linguistic discovery:

Excerpt 8: “I asked AI: ‘How can I say “Human touch (有人情味)” more formally?’ It suggested: ‘filled with the breath of life (充满生活气息).’ I paused… I never thought of that collocation. At that moment, I didn’t feel ‘it was better than me’; I realized I had found a new window of expression.” (P7)

For P7, AI Literacy shifted the focus from her “perceived failure as a heritage speaker” to a proactive learning process. As she noted in her diary (Excerpt 10), her anxiety decreased because she stopped asking “Why can’t I write well as a Chinese descendant?” and started seeing AI as a way to “practice formal register.” This shift from an identity-burden to functional-scaffolding is a key mechanism for reducing L2writing anxiety.

A recurring sub-theme was the emphasis on high-level monitoring and the “intentional rejection” of AI suggestions to maintain authenticity. This sense of ownership significantly enhanced writing engagement. P8 provided a sophisticated breakdown of the human-AI division of labor, emphasizing that the “final call” defines the author:

Excerpt 9: “AI contributes sentence optimization and grammar checks… but deciding what to delete and what to keep is me. I proactively delete some ‘fancy sentences’ because they don’t sound like me. This process makes me feel like the Author.” (P8)

Finally, the transition from “control” to “play” marked the highest level of engagement. When learners felt they could master the tool, AI provided “linguistic surprises” that fueled intrinsic interest. P3 described this as a shift from “following a standard” to “exploring possibilities”:

Excerpt 11: “I don’t see AI’s output as the ‘correct answer’ but as an example… It makes me feel like I am ‘playing with Chinese’ rather than losing control. It gives me new ideas, and I adapt them into my own words.” (P3)

These findings suggest that AI Literacy promotes engagement not by automating the writing process, but by providing a “safe space” for experimentation. By masking the “responsibility of error” and highlighting the “joy of discovery,” AI empowers learners to reclaim their agency in L2 writing.

## 5 Discussion

### 5.1 L2 Chinese writing AI literacy and L2 engagement: from technical operation to the control of writing procedure

Consistent with prior research, the present study found that L2 AI literacy positively predicted L2 engagement (*β* = 0.458). As a normative and authoritative discursive tool, AI-mediated correction provides an evaluative reference that enhances the perceived clarity of L2 writing standards (Barrot, 2023a). At the same time, such standardisation can release writers’ cognitive resources by offloading lower-level form-focused monitoring, thereby enabling a shift in attention toward constructing written texts in which linguistic form and meaning are more tightly integrated. During this process, AI provides timely feedback while supporting writers in making immediate generalisations about conventional language use, which strengthens perceived task competence (Lyu and Salam, 2025). When individuals can meet task demands and autonomously optimise the quality of their task outcomes, they can be more likely to clearly understand the nature, the requirements and desirable outcomes of the writing task, thereby letting out fears and stress in facing of unknowns in writing and building up confidence to organize writing, which signifies that they can self-responsible for their products and take control over the whole writing process. This pattern aligns with the competence component of CVT (Pekrun, 2006).

Building on enhanced competence, L2 writers are no longer positioned as passive recipients of AI’s normative pressure. Instead, they selectively retain, add to, or delete AI-generated suggestions in accordance with authentic communicative needs, exercising writer-centred value appraisal, value judgement and value adaptation, which means that students may not take themselves as writers but more of a creator who invests personal attitudes, personal insights and personal pursuits in their writing (Zhao and Wang, 2025). Through this process, behavioural engagement and cognitive engagement also improved, as endowing something with individualised traits and efforts potentially leading to a willingness to do more basic work and therefore guarantees that it can be worthwhile for potential readers (Akteruzzaman et al., 2023).

Furthermore, positive feedback derived from perceived linguistic progress—and subsequently reinforced by teacher feedback—may facilitate a motivational shift from the ought-to L2 self toward the ideal L2 self, thereby deepening emotional engagement in L2 writing (Dewaele and Li, 2021). Previous research likewise indicates that the ideal L2 self tends to be a stronger predictor of academic outcomes than the ought-to L2 self (Xu et al., 2024). For learners of Chinese heritage or those with Chinese as a heritage language, the ideal L2 self is often further articulated through the construction of an imagined identity, which foregrounds future-oriented self-imagery as a Chinese language user and strengthens beliefs that sustained effort will yield valued returns (Darvin and Norton, 2015).

### 5.2 A dual affective-regulatory pathway: the mediating role of anxiety and enjoyment

The findings of the present study indicate that AI literacy was found to be a significant predictor in reducing L2 writing anxiety (*β* = −0.643) and enhance L2 writing enjoyment (*β* = 0.482), thereby increasing L2 writers’ writing engagement (*β* = 0.231). Within CVT, L2 anxiety is typically understood as arising from appraisals of a high likelihood of failure and a low sense of control over task demands (Pekrun, 2006). Accordingly, when anxiety is salient, both L2 enjoyment (*β* = −0.337) and L2 engagement (*β* = −0.131) tend to be negatively affected (Horwitz et al., 1986; Botes et al., 2020).

In the present context, AI literacy appears to operate through a mechanism of normative feedback pressure: while the standardising force of AI feedback helps to reduce the cognitive burden associated with language monitoring, it simultaneously activates learner agency by motivating writers to actively appropriate and master linguistic rules (Bhusal, 2025). Once such rules are incorporated into their writing repertoire, writers are able to treat them as reliable criteria for sustaining writing quality. Building on this foundation, L2 writers can respond more effectively to topic and genre demands and evaluate whether AI-generated suggestions are compatible with their intended authorial voice and genre positioning (Barrot, 2023a). Moreover, the diverse linguistic options opened up through AI assistance may strengthen writers’ perceived control over their L2 texts, thereby reducing anxiety.

For beginners in L2 Chinese writing learning in our context, AI may have a stronger effect on alleviating L2 writing anxiety. Facing unfamiliar subjects tends to cause uncertainty, and, particularly in L2 writing, students encounter multiple academic pressures, including content, expressive, organisational, and logical thinking, and very fundamental language forms (Wang et al., 2025). All these, at the same time, occupy cognitive processing in the brain and may cause excessive cognitive load. AI, however, turns all uncertainties into practical advice with high accuracy, easing students’ cognitive challenges (Huang et al., 2025b). Besides, for heritage and ethnic Chinese learners in particular, AI literacy may further transform identity-related anxiety into a more manageable technical problem: writers’ concerns shift from “who I am” to “what I can do.” This reorientation foregrounds language resource construction rather than identity-based pressure to perform, enhancing perceived control and attenuating anxiety to some extent.

CVT also emphasises that L2 enjoyment primarily derives from positive appraisals of task value (Pekrun et al., 2009). AI literacy not only enables L2 writers to understand linguistic rules, but also supports them in applying such rules strategically. Through gradual exposure to evaluative norms and the aesthetic dimension of writing, learners may develop stronger recognition of the clarity and reliability of feedback-based evaluation practices (Wang et al., 2025). During corrective feedback episodes, AI can provide idiomatic Chinese expressions aligned with writers’ prompts, allowing learners to experience not only the instrumental value of AI tools but also the aesthetic and expressive richness of the Chinese language. When learners develop deep identification with task value—particularly intrinsic value—CVT predicts that they are more likely to seek greater authorial control, further explore underlying regularities in language use, and genuinely discover the intrinsic value of Chinese writing (Pekrun et al., 2002).

## 6 Conclusion

Adopting an explanatory sequential design, this study demonstrates that L2 Chinese Writing AI Literacy significantly and positively predicts L2 writing engagement, while also significantly and negatively predicting L2 writing anxiety. Moreover, L2 writing anxiety exerts significant negative mediating effects on both L2 writing enjoyment and L2 writing engagement, whereas L2 writing enjoyment shows a significant positive mediating effect on L2 writing engagement. Taken together, the findings suggest that L2 Chinese Writing AI Literacy strengthens writers’ behavioural and cognitive engagement primarily through learners’ increased perception of clarity and reliability of feedback-based standards, reduces anxiety by enhancing perceived control, and increases enjoyment through stronger appraisal of intrinsic task value. In turn, affective engagement further feeds back into behavioural and cognitive engagement, potentially forming a conceptually reinforcing mechanism.

Pedagogically, the results underscore the need for explicit instruction in how to use AI appropriately in L2 Chinese writing, with particular emphasis on critical AI literacy. Although the quantitative results indicate that participants’ overall AI literacy was at a moderately high level, there remains clear room for improvement. Diary and interview data revealed that some learners initially resisted AI use and were uncertain about how to engage with AI effectively. Accordingly, instruction should prioritise the cultivation of learners’ agency in human–AI collaboration. In particular, prompt-engineering training can help learners develop a principled dialogic mechanism with AI, rather than a one-way reception mode, thereby supporting a complementary and sustainable feedback ecology in which AI and teacher feedback function synergistically.

This study is among the first in L2 Chinese writing research to employ an explanatory sequential approach to investigate L2 Chinese Writing AI Literacy. Despite its novelty, several limitations should be acknowledged. First, although the quantitative phase included 408 learners, the sample predominantly represented the northern regions of China, which may limit the geographical representativeness of the findings. Second, the qualitative phase captured learners’ AI-assisted writing practices over only four weeks; therefore, the study may not fully account for longer-term trajectories and emerging patterns associated with sustained AI use. Third, while the qualitative findings suggest that reduced anxiety was closely linked to enhanced perceived control, this mechanism was not directly tested as a scalable mediator in the quantitative model. Future research should therefore expand sampling coverage, extend the observation period, and incorporate perceived control as an additional mediating variable to further clarify the affective pathway linking L2 Chinese Writing AI Literacy and L2 writing engagement.

## Data Availability

The datasets presented in this study can be found in online repositories. The names of the repository/repositories and accession number(s) can be found in the article/Supplementary material.
