# Supplementary material for: How AI literacy predicts L2 Chinese writing engagement: the mediating roles of anxiety and enjoyment
Source: Front Psychol. 2026 May 8;17:1811031. doi: 10.3389/fpsyg.2026.1811031 (PMC13195749; doi:10.3389/fpsyg.2026.1811031)
Supplement: Supplementary file 1 [file Supplementary_file_1.DOCX]

**Supplementary Material**

**Appendix A Questionnaire**

**A) L2 Chinese Writing AI Literacy**

1. I am familiar with various AI tools used in Chinese writing, such as grammar checkers and automatic translation tools.

2. I understand the specific functions that AI tools provide in Chinese writing, such as correcting grammatical errors and offering vocabulary suggestions.

3. I understand the advantages of using AI tools in Chinese writing, such as improving writing efficiency and reducing language errors.

4. I understand the limitations of AI tools in Chinese writing, such as their inability to understand context and the potential for generating inaccurate suggestions.

5. I can utilize AI tools to help expand my vocabulary and improve grammatical accuracy.

6. I can utilize AI tools to improve the logic and coherence of my writing.

7. I can utilize AI tools to better meet the requirements of writing tasks.

8. I can utilize AI tools to better meet the requirements of writing tasks.

9. I can utilize AI tools to improve my writing style, such as making my language more formal and my expressions more concise.

10. I can utilize AI-generated feedback to improve the quality of my Chinese writing.

11. I believe that AI feedback can help me improve my ability to evaluate my writing.

12. I am aware of the ethical issues involved in using AI to generate or edit Chinese writing, such as plagiarism and academic integrity.

13. I know that AI suggestions sometimes do not align with my writing style.

14. I am aware of the risks of sharing my writing data with AI tools, such as potential privacy breaches or data misuse.

15. I understand that over-reliance on AI for Chinese writing may hinder the improvement of my personal writing abilities.

16. I know that AI-generated text may differ from human-written content.

17. I find AI tools very helpful in organizing my writing thoughts.

18. I can utilize the feedback and suggestions from AI tools to improve the quality of my writing.

19. I can combine AI suggestions with human feedback to improve my writing skills.

20. I am confident in using various AI tools to improve my Chinese writing skills.

21. I trust AI suggestions and consider them equally important as feedback from human reviewers.

22. When receiving AI-generated feedback, I can remain calm and avoid feeling defensive.

23. AI tools have greatly improved my writing skills.

24. I am interested in using AI tools to assist my writing tasks.

25. I believe AI tools can provide faster and more effective writing suggestions than human reviewers.

26. I believe AI tools can reduce the stress of writing tasks and make the writing process more enjoyable.

**B) L2 Chinese Writing Enjoyment**

27. I enjoy expressing my thoughts in Chinese during the writing process.

28. I look forward to Chinese writing assignments.

29. I write Chinese essays because I enjoy the process.

30. I write in Chinese with interest.

**C) L2 Chinese Writing Anxiety**

31. I feel nervous and tense when writing in Chinese.

32. I worry about whether I can handle the various difficulties in Chinese writing.

33. I worry that I will not be able to express my thoughts clearly in Chinese.

34. I feel anxious when writing Chinese essays.

**D) L2 Chinese Writing Engagement**

35. When I encounter difficulties in Chinese writing, I concentrate until I solve them.

36. I put a lot of effort into my Chinese writing.

37. Even if the topic is difficult, I will continuously strive to improve my writing skills.

38. I pay great attention to detail when writing in Chinese.

39. I always get distracted or do other things when I should be writing.

40. I look forward to continuing to revise my Chinese writing drafts.

41. I enjoy learning new ways to express myself through Chinese writing.

42. I feel good when doing Chinese writing assignments.

43. I often feel frustrated when writing in Chinese.

44. I find Chinese writing assignments boring.

45. I carefully check my Chinese writing to ensure it accurately expresses my meaning.

46. In writing, I think about expressing an idea in different ways (e.g., words, structure).

47. I try to connect the suggestions from artificial intelligence with my previously learned Chinese knowledge.

48. I tried my best to understand the writing errors in the revision comments I received.

49. I simply completed the writing task perfunctorily without giving it any in-depth thought.

**Appendix B Stimulated Recall Diaries Questions**

**A) Week 1**

1 Which AI tools did you use this week (such as ChatGPT, DeepSeek, or a dictionary)? What specific problem did you use to solve?

2 When you first saw AI-generated Chinese content, what was your first reaction? Was it a feeling of "it's better than me" (loss of control/anxiety), or "it can help me" (control/pleasure)?

3 During use, did you worry that it was "cheating"? Did this worry affect your usage frequency?

**B) Week 2**

1. Of the suggestions the AI ​​gave this week, was there any that felt "off" or "didn't match what I meant"? How did you handle it?

2. For what you wanted to say (or the dialect/native language in your head), did the AI successfully translate it into "authentic written Chinese"? Did this process feel easy or confusing to you?

3. Do you feel you spent more time "thinking" or more time "copying and pasting" this week?

**C) Week 3**

1. Was there a moment this week that made you realize, "Writing essays is actually quite interesting"? Did the AI provide any brilliant expressions you would never have thought of yourself?

2. Have you tried sharing the AI-assisted writing with others (teachers, friends, social media)? How did you feel after receiving feedback?

3. Compared to the first week, has your anxiety changed? Is it because you understand AI better (improved literacy), or because you've gotten used to it?

**D) Week 4**

1. After this month, do you feel this essay was "written by yourself" or "written by AI"? How would you define your role in the writing process? (Was it planner, editor, or observer?)

2. If there were no teacher requirement, would you continue using AI to write Chinese next month? Why?

3. Has the AI tool changed your perspective on your identity as a "Chinese/heritage learner"? Has it helped you compensate for your weakness in reading and writing compared to your strong listening and speaking skills?

**Appendix C stimulated recall interviews**

1. Reflecting on these four weeks, if you compare your mindset during the first week of using AI writing with that of the last week, what was the biggest change? Was there a specific turning point that shifted your perspective on AI?

2. When encountering difficulties with AI (e.g., the Chinese it provided sounded strange), did you feel like you were 'unable to control the tool,' or 'able to correct it by rephrasing the question' (prompt)? How did this feeling of 'being able to correct it' affect your anxiety?

3. During these four weeks, was there any instance where the AI's feedback surprised you or made you realize, 'So Chinese can be said this way too'? Did this 'joy of discovery' make you invest more time in that task than usual?

4. Some say using AI makes people lazy (behavioral disengagement). But in your experience, did your brain work less or more to transform AI-generated content into 'your own writing'? Specifically, in what ways (e.g., checking word meanings, adjusting word order)?

5. Looking at your final draft from this week (showing the essay), who do you think is the 'author' of this article? Is it you? Is it AI? Or is it a product of your collaboration? If there was a 'collaboration,' what core value do you think you contributed?
